# Glucosinolates in Wild-Growing *Reseda* spp. from Croatia

**DOI:** 10.3390/molecules28041753

**Published:** 2023-02-12

**Authors:** Azra Đulović, Josip Tomaš, Ivica Blažević

**Affiliations:** 1 Department of Organic Chemistry, Faculty of Chemistry and Technology, University of Split, Ruđera Boškovića 35, 21000 Split, Croatia; 2 Institute de Chimie Organique et Analytique (ICOA), Université d’Orléans, UMR-CNRS 7311, BP 6759, F-45067 Orléans, France

**Keywords:** *Reseda alba*, *Reseda lutea*, *Reseda phyteuma*, glucosinolates, desulfoglucosinolates, 2-hydroxy-2-methylpropyl glucosinolate, glucoconringiin, UHPLC, MS/MS, NMR

## Abstract

Glucosinolates (GSLs) are a unique class of thioglucosides that evolved as defense mechanisms in the 16 families of the Brassicales order and present molecular tags which can be placed in a robust phylogenetic framework through investigations into their evolution and diversity. The GSL profiles of three Resedaceae species, *Reseda alba*, *R. lutea*, and *R. phyteuma*, were examined qualitatively and quantitatively with respect to their desulfo-counterparts utilizing UHPLC-DAD-MS/MS. In addition, NMR analysis of isolated 2-hydroxy-2-methylpropyl desulfoGSL (**d31**) was performed. Three Phe-derived GSLs were found in *R. lutea*, including glucotropaeolin (**11**) (0.6–106.69 mol g^−1^ DW), 2-(α-L-ramnopyranosyloxy)benzyl GSL (**109**) (8.10–57.89 μmol g^−1^ DW), glucolepigramin (**22**) (8.66 μmol g^−1^ DW in flower), and Trp-derived glucobrassicin (**43**) (0.76–5.92 μmol g^−1^ DW). The Phe-derived GSLs **109** (50.79–164.37 μmol g^−1^ DW), gluconasturtiin (**105**) (1.97 μmol g^−1^ DW), and **11** (tr), as well as the Trp-derived GSL glucobrassicin (**43**) (3.13–11.26 μmol g^−1^ DW), were all present in *R. phyteuma*. *R. alba* also contained Phe-derived **105** (0.10–107.77 μmol g^−1^ DW), followed by Trp-derived **43** (0.85–3.50 μmol g^−1^ DW) and neoglucobrassicin (**47**) (0.23–2.74 μmol g^−1^ DW). However, regarding the GSLs in *R. alba*, which originated from Leu biosynthesis, **31** was the major GSL (6.48 to 52.72 μmol g^−1^ DW) and isobutyl GSL (**62**) was the minor GSL (0.13 to 1.13 μmol g^−1^ DW). The discovered *Reseda* profiles, along with new evidence provided by GSL characterizations, were studied in the context of the current knowledge on GLSs in the Resedaceae family. With the exception of *R. alba*, the aliphatic GSLs of which were outliers among the Resedaceae species studied, this family typically contains GSLs derived primarily from Trp and Phe biosynthesis, which modifications resulted in GSLs unique to this family, implying presence of the specific genes. responsible for this diversification.

## 1. Introduction

There are ca. 96 species in the family Resedaceae Martinov, which are distributed predominantly in the Northern Hemisphere and a few Southern African countries [1,2]. Phylogenetic analysis of internal transcribed spacers of the nuclear ribosomal DNA and plastid *trnL*-*trnF* sequences of 66 species from all genera of the Resedaceae confirmed its traditional subdivision into three tribes: two monophyletic genera (*Caylusea* and *Sesamoides*) and one natural group (core *Reseda*), which includes four genera (*Ochradenus, Oligomeris, Randonia,* and *Reseda*). Four out of six taxonomic sections within *Reseda* (*Leucoreseda*, *Luteola*, *Glaucoreseda*, and *Phyteuma*) are monophyletic in origin [3]. Crown-group Resedacae, which includes *Reseda* genus, (represented by 68 species), has been dated to (13.5-)12.6, 10.5(−8.7) million years ago (Ma) [1,2,4]. Many species of *Reseda* are restricted to the Mediterranean Basin, while four species, *R. alba*, *R. lutea*, *R. luteola,* and *R. phyteuma*, are distributed worldwide [3]. There are five spp. of *Reseda* genus (mignonettes) known to be wild-growing in Croatia, i.e., *Reseda alba* L. (white mignonette), *R. lutea* L. (yellow or wild or cutleaf mignonette), *R. luteola* L. (dyer’s rocket; weld), *R. phyteuma* L. (garden mignonette, common mignonette), and the critically endangered *R. inodora* Rchb. [5].

Decisive species identifications, the availability of trustworthy phylogenies, and conclusive chemical analyses are conditions of utmost relevance in exploring the evolution of any class of metabolites [6]. It is generally accepted that genes for secondary metabolites, including GSLs, are inherited due to the evolutionary advantages they impart to the plant, especially for defense against abiotic stress, plant pathogens, parasites, and herbivores [7,8]. There are more than 130 distinct GSLs produced by Brassicales species, while the structures of only 90 glucosinolates (GSLs) have been confirmed using MS and NMR to date [9]. Structural variation in intact GSLs is achieved through the use of different amino acid precursors, including methionine (Met), alanine (Ala), valine (Val), leucine (Leu), isoleucine (Ile), glutamic acid (Glu), tyrosine (Tyr), phenylalanine (Phe), and tryptopane (Trp), and the sequential modification of side chains. The genetic mechanisms governing GSL biosythesis in the model plant *Arabidopsis thaliana*, and to some extent in *Brassica* spp., are well understood and are useful for investigating the underlying mechanisms of GSL production at a very fundamental level. Novel GSLs and the genes that encode the proteins that control their biosynthesis pathways outside of these plant species could significantly improve our understanding of the phytochemistry, evolution, and natural history of Brassicales [7].

The progenitors of *Brassica* (mustards and cabbage) and similar plants evolved GSLs as a chemical defense over 90 Ma [10]. When they first emerged 92 Ma ago, Brassicales could only produce GSLs from phenylalanine and branched-chain amino acids. Indolic GSLs, which are produced from the amino acid tryptophan, first occurred 77.5 Ma after the At-β whole-genome duplication event (95% highest posterior density, HPD, 42–112 Ma). A second significant phase of escalation took place when the ancestors of the plant lineages Capparaceae and Cleomaceae produced a new set of GSLs derived from methionine, another novel substrate. The final escalation event appeared 32 Ma (95% HPD 17–46 Ma) with the evolution of Brassicaceae (the mustard family), which contains the greatest diversity of GSLs within Brassicales [10].

To date, some important studies on the morphology, anatomy, palynology, cytogenetics, pharmacology, and phytochemistry of the family Resedaceae have been conducted [3,4,10]. For this study, three *Reseda* species—*R. alba*, *R. lutea*, and *R. phyteuma*—were collected. *R. alba* is a well-liked ornamental plant on account of its spike-like racemes of fragrant white flowers, although in some parts of Italy and Greece its young leaves and flowering branches have been traditionally used as wild vegetables [11]. Since the first millennium BC, *R. lutea* leaves and flowers have been used to manufacture a yellow dye known as “weld,” primarily in the form of the flavonoid luteolin, even though a related plant, *R. luteola*, has more frequently been used for that purpose [12]. *R. phyteuma*, having a taste similar to cabbage, is used as a potherb in Greece [13]. Pharmacological studies of extracts from the investigated *Reseda* species have revealed great biological potential, including cytotoxic, analgesic, anti-inflammatory, antibacterial, and antioxidant activities [14,15,16,17,18,19,20,21]. Resedaceae plants are known to contain GSLs in their tissues, just like all the other families in the order Brassicales. GSLs, through their breakdown products isothiocyanates, are extremely harmful to most insects, and give mustards their pungent flavors, appreciated by humans, and have been investigated for their diversified and generally marked bioactivites, especially anticancer activities [22,23]. Table 1 displays the distribution of GSLs in plants of the Resedaceae family that have been the subject of research to date.

Qualitative analyses of GSLs in Resedaceae published up to 2001 were reviewed by Fahey et al. [24]. Bennett et al. (2004) reported a quantitative analysis of arylaliphatic and indolic GSLs in the seeds of *Reseda luteola* and *R. odorata*, while the *O*-glycosylated GSL, 2-(*α*-L-rhamnopyranosyloxy)benzyl GSL (**109**) was found only in *R. lutea* (25.0–50.0 µmol g^−1^ dry weight, DW). Outside of the genus *Reseda*, GSL **109** was found in the plant *Ochradenus baccatus*, with 7.0 µmol g^−1^ DW in the root [36]. Two arylaliphatic GSLs, glucobarbarin (**40*S***) and epiglucobarbarin (**40*R***), were identified in *R. luteola*, with contents ranging from 0.1 to 10.0 and 25.0 to 50.0 µmol g^−1^ DW, respectively. In the same work, in the seeds of *Caylusea abyssinica*, in addition to the same arylaliphatic GSLs, gluconasturtiin (**105**) and **40*R*** were found to have the highest contents (10.0–25.0 µmol g^−1^ DW) [25]. The presence of **40*S***, a characteristic GSL in the genus *Barbarea* (Brassicaceae), was confirmed also in *R. luteola*, despite the great evolutionary distance between them. Agerbirk et al. (2021) investigated the same plant and determined the presence of **40*R***, which in the seeds accounted for 5% of the total amount of enantiomeric glucobarbarins, i.e., 1% in the leaf. In addition, the analysis also revealed significant levels of the apparent hydroxybutyl GSL, which could be either Met-derived 4-hydroxybutyl GSL ([26]) (unknown from basal families at the time) or Leu-derived 2-hydroxy-2-methylpropyl GSL (glucoconringiin, **31**) [17]. GSL **40*S*** was identified as the main GSL in the leaf of *R. luteola* and accounted for over 90% of the total GSLs from the leaf surface (0.5 µmol g^−1^ of fresh plant material) [22,32]. The indole GSL glucobrassicin (**43**) was found in the seeds of *R. odorata* in the range of 10.0–25.0 µmol g^−1^ DW [25].

GSLs identified in *R. alba* included plant aliphatic GSLs, not common in the Rese-daceae family, i.e., hydroxyaliphatic GSL glucoconringin (**31**) and (2*S*)-2-hydroxybut-3-enyl GSL (epiprogoitrin), as well as two arylaliphatic GSLs, glucosinalbin (**23**) and **105**, and indole GSLs **43** and **47** [24,26,27,28].

2-(*α*-l-Arabinopyranosyloxy)-2-phenylethyl GSL was the first identified extraglycosylated GSL containing arabinose as an additional carbohydrate moiety. It was isolated from the plant *Sesamoides interrupta* and was also found in a plant of another genus, *R. phyteuma* [35]. Recently, another arabinosylated GSL, 2′′-*O*-(*α*-l-arabinopyranosyloxy)benzyl GSL (**158**), was found in the roots of the desert plant *Ochradenus baccatus* (4.1 µmol g^−1^ DW), representing an additional genus in the Resedaceae family in which these specific GSLs were identified [36].

The purpose of this work was to identify and quantify GSLs in different plant parts of three *Reseda* species that are wild-growing in Croatia utilizing their desulfo-counterparts using UHPLC-DAD-MS/MS in order to understand the Resedaceae family’s biosynthetic potential. Additionally, 2-hydroxy-2-methylpropyl GSL (glucoconringiin, **31**), which was thoroughly characterized by means of MS^2^ and NMR, as a desulfated derivative, was isolated and purified from flowers, which were found to be the best source for this uncommon GSL. By examining different plant parts, the existing GSL profiles of *R. alba*, *R. lutea*, and *R. phyteuma* were expanded. Finally, the revised GSL profiles were evaluated in terms of their biosynthetic characteristics and evolution.

## 2. Results and Discussion

In this study, three *Reseda* plant species wild-growing in Croatia, *R. alba*, *R. lutea*, and *R. phyteuma*, were investigated. According to UHPLC-DAD-MS/MS analysis, *R. lutea* and *R. phyteuma* showed comparable GSL profiles, while that of *R. alba* was completely different (Figures S1–S3 and Table 2). The MS^2^ spectra are given in Figures S4A,B and S5A–D.

**Table 2:** Quantitative analysis of GSLs in individual plant organs of researched plants of the genus *Reseda*.

No. *	Identified Glucosinolate	*t_R_* (min)	[M + Na]^+^	Plant Tissue (μmol g^−1^ DW)			
*Reseda alba*				Flower	Leaf	Stem	Root
Leu-derived							
**31**	Glucoconringiin	1.64	334	52.72 ± 2.22	6.48 ± 0.51	25.29 ± 1.13	19.70 ± 1.89
**62**	Isobutyl GSL	5.30	318	1.00 ± 0.24	0.13 ± 0.04	0.13 ± 0.05	1.13 ± 0.22
Phe-derived							
**105**	Gluconasturtiin	7.93	366	n.d.	0.10 ± 0.03	1.20 ± 0.37	107.77 ± 2.83
Trp-derived							
**43**	Glucobrassicin	7.21	391	1.64 ± 0.32	3.50 ± 0.11	0.85 ± 0.10	1.69 ± 0.67
**47**	Neoglucobrassicin	9.34	421	0.32 ± 0.08	0.23 ± 0.02	0.55 ± 0.11	2.74 ± 0.38
*Reseda lutea*				Flower	Leaf	Stem	Root
Phe-derived							
**22**	Glucolepigramin	5.22	368	8.66 ± 1.00	n.d.	n.d.	n.d.
**11**	Glucotropaeolin	6.51	352	1.64 ± 0.76	0.6 ± 0.09	5.67 ± 0.75	106.69 ± 3.04
**109**	2-(*α*-L-Ramnopyranosyloxy)-benzyl GSL	6.78	514	57.89 ± 3.19	20.50 ± 1.50	14.86 ± 1.86	8.10 ± 1.06
Trp-derived							
**43**	Glucobrassicin	7.21	391	5.92 ± 0.34	0.76 ± 0.15	2.45 ± 0.25	3.26 ± 0.17
*Reseda phyteuma*				Flower	Leaf	Stem	Siliquae
Phe-derived							
**11**	Glucotropaeolin	6.51	352	n.d.	n.d.	tr	n.d.
**109**	2-(*α*-L-Ramnopyranosyloxy)-benzyl GSL	6.78	514	150.84 ± 3.52	164.37 ± 3.72	50.79 ± 1.23	123.93 ± 2.64
**105**	Gluconasturtiin	7.93	366	n.d.	n.d.	1.97 ± 0.21	n.d.
Trp-derived							
**43**	Glucobrassicin	7.21	391	8.47 ± 0.18	4.79 ± 0.73	11.26 ± 0.77	3.13 ± 0.12

**Figure 1:**
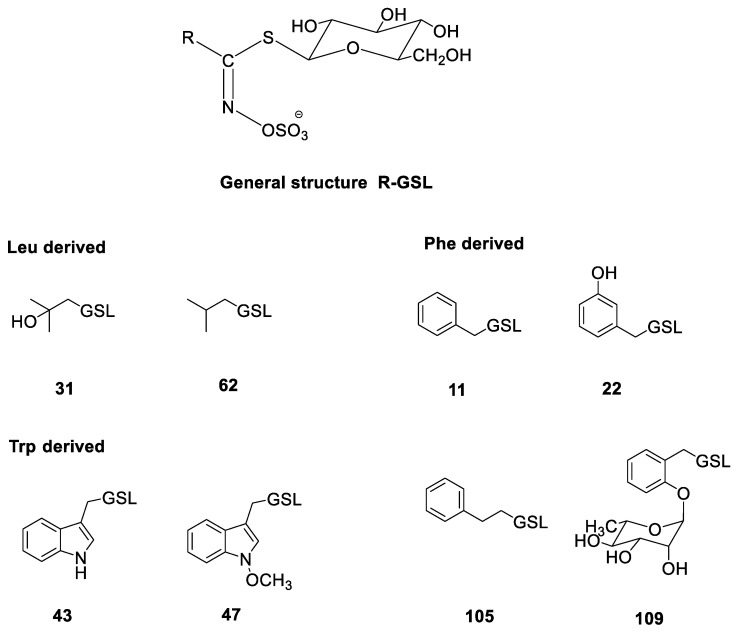
Structures of the GSLs identified in the investigated *Reseda* sp. (cf. Table 2): benzyl GSL (glucotropaeolin, **11**); 3-hydroxybenzyl GSL (glucolepigramin, **22**); 2-hydroxy-2-methylpropyl GSL (glucoconringiin, **31**); indol-3-ylmethyl GSL (glucobrassicin, **43**); *N*-methoxyindol-3-ylmethyl GSL (neoglucobrassicin, **47**); 2-phenylethyl GSL (gluconasturtiin, **105**); 2-(*α*-L-ramnopyranosyloxy)benzyl GSL (**109**). Numbering system is related to the GSL numbers given in the review paper by Blažević et al. [9].

* No.—Numbering system is related to the glucosinolate numbers given in the review paper by Blažević et al. [9]. The structures are shown in Figure 1. All chromatograms are given in Figures S1–S3, while MS^2^ spectra are given in Figure S4A,B. [M + Na]^+^—sodium adduct of desulfoglucosinolate; *t*_R_—retention time at the UHPLC-DAD-MS/MS conditions reported here; GSL—glucosinolate; tr—traces; n.d.—not detected; DW—dry weight of plant material. Data are expressed as mean values ± standard errors (*n* = 3).

Arylaliphatic, indolic, and O-glycosylated GSLs were identified in *R. lutea*. All plant tissues contained GSLs **11**, **43**, and **109**. In addition, it was observed that as the content of **11** decreased, the content of **109** increased in each individual tissue. The highest concentration of **11** was found in the root, with 106.69 µmol g*^−^*^1^ DW, while the highest concentration of **109** was found in the flower, with 57.89 µmol g*^−^*^1^ DW. GSL **109** is an isomer of 4-(α-L-ramnopyranosyloxy)benzyl GSL (glucomoringin, **110**), which is a hallmark of another family, Moringaceae. In addition to having different retention times, **d109** and **d110** vary in their MS^2^ spectra [6]. Figure S4B shows the MS^2^ spectra of **d109** at collision energies of 20 and 30 V. Typical thioglucosidic bond fragmentation results in [anhydroglucose + Na]^+^ at *m*/*z* 185 (type **a**) and [M-162 + Na]^+^ at *m*/*z* 219 (type **b**), while the type **c** fragment results from loss of an anhydroglucose, [M-162 + Na]^+^ [37]. Fragments from the elimination of anhydrorhamnose, anhydroglucose, and thioglucose (fragment **h**) were observed, with the characteristic fragment *m*/*z* 334 resulting from the loss of glucose (*m*/*z* 180). Glucolepigramin (**22**) was identified only in the flower (8.66 µmol g*^−^*^1^ DW) using desulfoglucolepigramin as a standard isolated from *Lepidium graminifolium* [38], corroborating a recent discovery [29].

*O*-Rhamnosylated **109** and indole **43** GSLs were identified in all parts of the *R. phyteuma* plant. The content of **109** was significantly higher than in *R. lutea*, up to 164.37 µmol g*^−^*^1^ DW in the flower, which is why this plant species represents a good source of this GSL. Arylaliphatic **11** and **105** were identified only in the stem and at very low concentrations.

**Figure 2:**
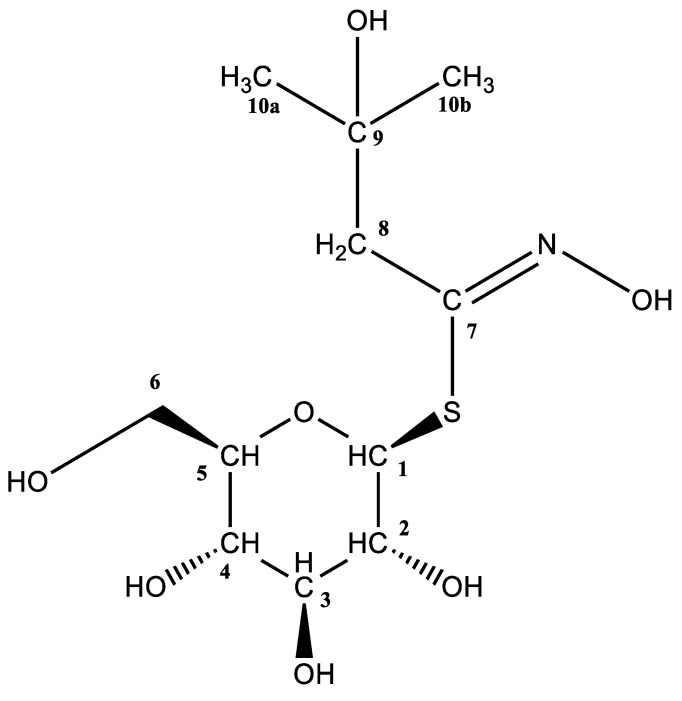
Structure of 2-hydroxy-2-methylpropyl desulfoglucosinolate (desulfoglucoconringiin, **d31**).

*R. alba* had a markedly different GSL profile compared to the other two species of this family that have been studied. The dominant desulfoglucosinolate (dGSL) signal at *t*_R_ = 1.64 min and *m*/*z* 334 was assumed to be 2-hydroxy-2-methylpropyl GSL (glucoconringiin, **31**) based on a previous report by Olsen and Sorensen (1979) [27]. The MS^2^ spectrum (Figure S4A) revealed characteristic fragments, the origins of which were previously explained (**a**, **b**, and **c** fragments), and a fragment specific to hydroxylated aliphatic dGSLs that originated from water loss (type **f**, *m*/*z* 316). As no standard was available, the GSL was isolated from the flower due to its high amount (52.72 µmol g*^−^*^1^ DW), and NMR spectra of its desulfo-counterpart were recorded (Figure 2 and Figure S5A–D). The H-1/H-2 and H-5/H-6a/H-6b spin systems’ coupling constants correspond to a thioglucosyl moiety. Additionally, two distinct doublets were found between two H-8 protons (^2^J = 15.2 Hz) at 2.83 and 2.74 ppm, respectively. Furthermore, two singlets at 1.35 and 1.33 ppm, respectively, denote two methyl groups (H-10a and H-10b). Finally, according to ^13^C NMR, the same methyl groups are represented by two peaks at 28.5 and 27.9 ppm, and the precise quaternary carbon that those methyl groups are next to may be seen at 71.1 ppm.

The *m*/*z* 318 signal in MS^2^ (Figure S4A) may correspond to two isomers, desulfoglucocochlearin (**d61**) and isobutyl dGSL (**d62**). This GSL was previously identified in *Sisymbrium officinale* at the same retention time (t_R_ = 5.30 min), which is consistent with this study [37].

In evolutionary terms, Brassicales were initially only able to synthesize GSLs from Phe and branched-chain amino acids, after which the indolic GSLs, which are produced from the amino acid Trp, started to appear [10]. As can be seen from Table 1 and from the results obtained in this study, the GSLs of Resedaceae are mostly biosynthesized from Phe/Tyr and Trp. The only indole-type GSLs appear to be **43** and **47**. The distribution of side-chain-modified Trp-derived GSLs in basal families is poorly understood, with Salvadoraceae and Tovariaceae as exceptions, giving some reason to believe that *N*-methoxylation is rather ancient [6,39]. In the case of Phe GSLs, biosynthetic diversification occurred during evolution. Glucotropaeolin (**11**) (*R. lutea* and *R. phyteuma*) originated directly from Phe biosynthesis, which can be further hydroxylated to produce **22** (*R. lutea*). The GSL **109**, a Phe-derived GSL additionally glycosylated in the ortho position and with a rhamnose moiety, as a tag for the Resedaceae family, was found in high amounts in *R. lutea* and *R. phyteuma*. Furthermore, another Phe-derived GSL with an arabinose moiety was recently reported in *Ochradenus baccatus*, implying that plants in the Resedaceae have specific genes responsible for the glycosylation of the benzylic ring in the ortho position, which needs to be investigated further. Additionally, other GSLs are biosynthesized after the elongation of Phe into homoPhe, which is a precursor of gluconasturtiin (**105**) found in high amounts in the root of *R. alba* (107.77 µmol g*^−^*^1^ DW) and only in traces in the stem of *R. phyteuma*. This GSL, after hydroxylation, can produce epiglucobarbarin (**40R**) and glucobarbarin (**40S**), which were not detected in our study, although they were previously reported elsewhere. Further modification of these GSLs can produce 2-(*α*-L-arabinopyranosyloxy)-2-phenylethyl GSL (**4**), another exotic GSL, previously reported in *R. phyteuma* and *Sesamoides interrupta*, originating from homoPhe biosynthesis (Table 1).

*R. alba* seems to diverge from the other investigated species in Resedaceae with respect to GSL chemistry, as aliphatic GSLs have additionally been detected only in *R. luteola* [6]. Isobutyl GSL (**62**), found in all plant parts, is biosynthesized from Leu, which is a precursor of glucocorningiin (**31**), and was found in all investigated plant parts. This GSL is found in numerous plant families, including most members of the Brassicaceae (such as *Cochlearia* spp., *Conringia orientalis*, *Arabis procurrens*, *Draba aizoides*, etc.), but also the Akaniaceae (*Bretschneidera sinensis*), the Limnanthaceae (*Limnanthes* spp.), and the Tropaeolaceae (*Tropaeolum peregrinum*) [24,25,40]. In terms of side-chain modification, *β*-hydroxylation of aliphatic GSLs is considered to be ancient [39].

## 3. Materials and Methods

### 3.1. Materials and Reagents

All plant samples were collected from plants wild-growing in Croatia in April 2021. *Reseda alba* L., *R. lutea* L., and *R. phyteuma* L. samples were collected in Split (43°30’7″ N, 16°29′8″ E), Split (43°30’31″ N, 16°23’30″ E), and Tisno (43°48’34″ N, 15°37’45″ E), respectively. The specimen vouchers were stored under numbers ZOKRA1, ZOKRL1, and ZOKRF1. Sinigrin, DEAE-Sephadex A-25 (GE Healthcare), and sulfatase (type H-1 from Helix pomatia) were purchased from Sigma-Aldrich (St. Louis, MO, USA); glucotropaeolin (**11**), glucobrassicin (**43**), *N*-methoxyglucobrassicin (**47**), and gluconasturtiin (**105**) were obtained from Phytoplan Diehm & Neuberger GmbH (Heidelberg, Germany); while glucolepigramin (**22**) was previously isolated from *L. graminifolium* [38]. All other chemicals and reagents were of analytical grade.

Commercial sulfatase requires additional purification steps. Ultrapure water (30 mL) and 10 kU of aryl sulfatase were mixed with absolute ethanol (30 mL). The mixture was centrifuged for 20 min at room temperature at 2650× *g*. The supernatant was mixed with ethanol (90 mL). The mixture was further centrifuged for 15 min at room temperature at 1030× *g*, after which supernatants were removed and discarded. The combined pellets were dissolved in ultrapure water (25 mL) and thoroughly vortexed, dispensed into 1 mL tubes, and frozen (−20 °C).

### 3.2. Isolation and Chemical Analysis

#### 3.2.1. Isolation of Desulfoglucosinolates

GSLs were extracted from different plant parts, as previously reported [37]. To inactivate the endogenous myrosinase, the plant material was ground into a fine powder, and 100 mg was extracted for 5 min at 80 °C in 2 mL MeOH/H_2_O (70:30 *v*/*v*). Each extract was loaded onto a mini-column containing 0.5 mL of DEAE-Sephadex A-25 anion-exchange resin and conditioned with 25 mM acetate buffer (pH 5.6). To achieve the best desulfation conditions, buffer solution was added to the column after it had been washed with 70% MeOH and 1 mL of ultrapure water. Purified sulfatase at an amount of 20 µL (0.35 U/mL) was placed into each mini-column and allowed to stand for 18 h at room temperature. The dGSLs were then eluted with 1.5 mL of ultrapure H_2_O, lyophilized, and diluted to 1 mL. The samples were kept at −20 °C until they underwent UHPLC-DAD-MS/MS analysis.

#### 3.2.2. UHPLC-DAD-MS/MS Analysis

UHPLC-DAD-MS/MS (Ultimate 3000RS with a TSQ Quantis MS/MS detector, Thermo Fisher Scientific, MA, USA) and a Hypersil GOLD C18 column (3.0 µm, 3.0 × 100 mm, Thermo Fisher Scientific, MA, USA) were used for the analysis. A gradient consisting of solvent A (50 μM NaCl in H_2_O) and solvent B (acetonitrile:H_2_O 30:70 *v*/*v*) was applied at a flow rate of 0.5 mL/min as follows: 0.14 min 96% A and 4% B; 7.84 min 14% A and 86% B; 8.96 min 14% A and 86% B; 9.52 min 5% A and 95% B; 13.16 min 5% A and 95% B; 13.44 min 96% A and 4% B; 15.68 min 96% A and 4% B. The injection volume was 5 µL, and the column temperature was maintained at 25 °C. The electrospray interface was an H-ESI source operating at 350 °C with 3.5 kV of capillary voltage. The ion-transfer tube was set at 325 °C. The system was operated in the positive ion mode with a mass range of *m*/*z* 150–800, a scan rate of 1000 (Da/sec), and a resolution of 0.4 (FWHM). Nitrogen was used as: sheath gas set at 5.58 L/min, aux gas at 7.97 L/min, and sweep gas at 1.5 L/min. MS^2^ parameters included Q1 resolution 0.4 (FWHM), Q3 resolution 0.4 (FWHM), and CID Gas 1.5 (mTorr). MS^2^ analysis of each visually detected peak was performed with a systematic search for *m*/*z* values of dGSL Na^+^ adducts, along with characteristic MS^2^ fragments (described in the Supplementary Materials). The signals were recorded at 227 nm with a DAD detector. Peaks of GSLs were quantified from UV peak areas using a calibration curve of pure desulfosinigrin solution with a concentration range from 0.14 to 1.4 mM (R^2^ = 0.98, y = 0.019x+0.997) and response proportionality factors (RPFs) for each individual dGSL. The following RPF values were used to quantify dGSLs: 0.29 for **43**; 0.20 for **47**; 0.95 for **11** and **105** [41]; and an arbitrary RPF of 1.0 for aliphatic GSLs **22**, **31**, **62**, and **109 [42]**.

#### 3.2.3. NMR Mesurements

NMR spectra were recorded using a Bruker AV600 spectrometer (Bruker BioSpin GmbH, Rheinstetten, Germany) with a 5 mm diameter probe and z-gradient accessories at 25 °C. The ^1^H (zg30) and ^13^C{1H} (zgpg30) NMR spectra were recorded at 600.130 and 150.903 MHz, respectively. The chemical shifts (δ/ppm) of the ^1^H spectra were referenced to the D_2_O signal (^1^H: δ = 4.80 ppm), and those of the ^13^C spectra were referenced to 1,4 dioxane d_8_ (^13^C: δ = 66.66 ppm), which was used as an external reference. The ^1^H spectra were recorded with the following parameters: sweep width of 20.0 ppm, transmitter frequency offset of 9.0 ppm, FID resolution of 0.37 Hz, relaxation delay of 1 s, acquisition time of 1.36 s, and 64 scans. The ^13^C spectra were acquired using the following parameter values: sweep width of 240.0 ppm, transmitter frequency offset of 100.0 ppm, FID resolution of 0.55 Hz, relaxation delay of 1 s, acquisition time of 0.91 s, and 128 scans during 64 loop counts. The assignment of ^1^H and ^13^C signals in the NMR spectra was confirmed by cross peaks in the ^1^H-^1^H COSY (correlation spectroscopy) and ^1^H-^13^C HSQC (heteronuclear single-quantum coherence) 2D spectra. The COSY (cosygpqf) with a standard π/2 pulse sequence was measured with 2048 points in dimension F2 and 512 increments in dimension F1. The latter was subsequently zero-filled to 1024 points. The increments were obtained with 4 scans each, a 16.00 ppm spectral width, and a relaxation delay of 1.0 s. The FID resolution was 4.69 Hz/point and 37.51 Hz/point in the F2 and F1 dimensions, respectively. HSQC spectra (hsqcedetgppsisp.2) were recorded with 2048 points in the F2 dimension and 256 increments in the F1 dimension, subsequently zero-filled to 1024 points. For each increment, 32 scans were collected using a relaxation delay of 1.0 s. Spectral widths were 15.00 ppm (F2) and 180.0 ppm (F1), with corresponding resolutions of 8.78 and 212.22 Hz/point in the F2 and F1 dimensions, respectively.

2-Hydroxy-2-methylpropyl desulfoglucosinolate (desulfoglucoconringiin, **d31**): ^1^H NMR (600 MHz, D_2_O) *δ* (ppm) = 5.20 (d, *^3^J* = 9.9 Hz, 1H, H-1), 3.93 (dd, *^2^J*_6a-6b_ = 12.6 Hz, *^3^J*_6a-5_ = 2.2 Hz, 1H, H-6a), 3.73 (dd, *^2^J*_6b-6a_ = 12.6 Hz, *^3^J*_6b-5_ = 6.0 Hz, 1H, H-6b), 3.61–3.55 (m, 2H, H-3, H-5), 3.50–3.44 (m, 2H, H-2, H-4), 2.83 (d, *^2^J* = 15.2 Hz, 1H, H-8a), 2.74 (d, *^2^J* = 15.2 Hz, 1H, H-8b), 1.35 (s, 3H, H-10a), 1.33 (s, 3H, H-10b); ^13^C NMR (151 MHz, D_2_O) *δ* (ppm) = 152.7 (C=N), 81.9 (C-1), 80.0 (C-5), 77.1 (C-3), 72.3 (C-2), 71.1 (Cq), 69.3 (C-4), 60.7 (C-6), 43.7 (C-8), 28.5 (C-10a), 27.9 (C10-b).

## 4. Conclusions

The investigation of GSL profiles of *Reseda* spp., as well as a review of the literature and an experimental study of other species, enabled a biosynthetic characterization of the Resedaceae family. Advances in our understanding of GSL biosynthesis and its evolution in Resedaceae species would benefit further from molecular genetic investigations. The existence of specific extraglycosylated GSLs, i.e., 2′′-*O*-(*α*-L-arabinopyranosyloxy)benzyl GSL (**158**) and 2-(α-L-arabinopyranosyloxy)-2-phenylethyl GSL (**4**) bearing arabinose and 2-(*α*-L-ramnopyranosyloxy)benzyl GSL (**109**) bearing rhamnose, suggests that specific genes are responsible for the evolution of these GSLs. However, more species should be investigated in order to chemically relate this family to other families that contain GSLs as chemical tags, as our and previous studies on *R. alba* suggest diversity in the genus itself. Appropriate analytical methods allowed the identification of previously unknown Leu-derived isobutyl GSLs. This study encourages further research into the relationship between GSLs as chemical tags of 16 families and corresponding phylogenetic investigations of aspects of their evolution.

## Figures and Tables

**Table 1:** Distribution of glucosinolates in plants belonging to the Resedaceae family investigated to date.

Amino Acid Precursor	Met	Leu		Phe/Tyr								Trp		References
No. *	24*S*	31	4	11	22	23	40*S*	40*R*	105	109	158	43	47	
***Caylusea abyssinica***						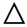	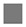	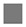	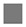					[24,25]
***Reseda alba***	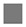	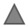				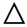	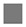		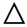			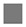	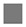	[24,26,27,28]
***R. complicata***												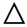	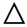	[24]
***R. crystallina***												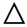		[24]
***R. lutea***				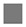	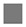	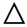				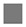		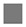		[14,24,25,29]
***R. luteola***						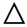	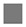	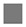	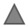			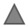		[6,24,25,27,30,31,32,33]
***R. media***				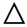	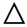				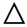			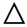		[24,34]
***R. odorata***							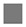	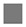		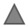		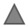		[6,24,25,27,30]
***R. phyteuma ^1^***			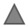			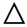	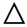	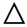	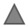	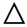		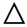		[24,30,35]
***R. stricta***						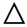			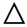	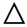				[24,30]
***R. suffruticosa***									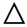					[30]
***Sesamoides interrupta ^2^***			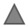				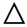	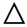	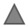					[24,35]
***Ochradenus baccatus***				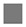			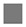		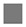	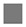	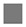	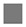		[36]

* No.—Numbering system is related to the GSL numbers given in a review paper by Blažević et al. [9]. ^1^ Syn. *Sesamoides pygmaea*; ^2^ Syn. *S. canescens* and *R. canescens*; 2-(α-L-arabinopyranosyloxy)-2-phenylethyl GSL (**4**); benzyl GSL (glucotropaeolin, **11**); 3-hydroxybenzyl GSL (glucolepigramin, **22**); 4-hydroxybenzyl GSL (glucosinalbin, **23**); (2*S*)-2-hydroxybut-3-enyl GSL (epiprogoitrin); 2-hydroxy-2-methylpropyl GSL (glucoconringiin, **31**); (2*S*)-2-hydroxy-2-phenylethyl GSL (glucobarbarin, **40*S***); (2*R*)-2-hydroxy-2-phenylethyl GSL (epiglucobarbarin, **40*R***); indol-3-ylmethyl GSL (glucobrassicin, **43**); *N*-methoxyindol-3-ylmethyl GSL (neoglucobrassicin, **47**); 2-phenylethyl GSL (gluconasturtiin, **105**); 2-(α-L-rhamnopyranosyloxy)benzyl GSL (**109**); 2′′-*O*-(*α*-L-arabinopyranosyloxy)benzyl GSL (**158**). 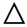-“Circumstantial evidence”—reasonable but not conclusive evidence of qualitative analysis; 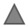-“Present”—qualitative analysis performed using relevant analytic methods (standards, MS, and NMR); 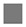-Qualitative and quantitative analyses performed.

## Data Availability

Not applicable.

## Supplementary Materials

The following are available online at https://www.mdpi.com/article/10.3390/molecules28041753/s1, Figure S1: Chromatogram of desulfoglucosinolates obtained from the different plant parts of *Reseda alba* (flower, leaf, stem, and root): d31—desulfo-2-hydroxy-2-methylpropyl dGSL (desulfoglucoconringiin), d43—desulfoindol-3-ylmethyl dGSL (desulfoglucobrassicin), d47—desulfo-*N*-methoxyindol-3-ylmethyl dGSL (desulfoneoglucobrassicin), d62—desulfoisobutyl dGSL, d105—desulfo-2-phenylethyl dGSL (desulfogluconasturtiin). Figure S2: Chromatogram of desulfoglucosinolates obtained from the different plant parts of *Reseda lutea* (stem, flower, leaf, and root): d11—desulfobenzyl dGSL (desulfoglucotropaeolin), d22—desulfo-3-hydroxybenzyl dGSL (desulfoglucolepigramin), d43—desulfoindol-3-ylmethyl dGSL (desulfoglucobrassicin), d109—desulfo-2-(α-L-rhamnopyranosyloxy)benzyl dGSL. Figure S3: Chromatogram of desulfoglucosinolates obtained from the different plant parts of *Reseda phyteuma* (flower, leaf, stem, and siliquae): d11—desulfobenzyl dGSL (desulfoglucotropaeolin), d43—desulfoindol-3-ylmethyl desulfoGSL (desulfoglucobrassicin), d105—desulfo-2-phenylethyl dGSL (desulfogluconasturtiin), d109—desulfo-2-(α-L-rhamnopyranosyloxy)benzyl dGSL. Figure S4: (A). MS2 spectra at 15V of detected desulfoglucosinolates. (B). MS2 spectra of d109 at 20V and 30 V. Figure S5: (A). ^1^H NMR spectrum of desulfo-2-hydroxy-2-methylpropyl dGSL (desulfoglucoconringiin). (B). ^13^C NMR spectrum of desulfo-2-hydroxy-2-methylpropyl dGSL (desulfoglucoconringiin). (C). COSY spectrum of desulfo-2-hydroxy-2-methylpropyl dGSL (desulfoglucoconringiin). (D). HSQC spectrum of desulfo-2-hydroxy-2-methylpropyl dGSL (desulfoglucoconringiin).

## Sample Availability

Samples of the compounds are available from the authors.
