# Supplementary material for: Glucosinolates in Wild-Growing Reseda spp. from Croatia
Source: Molecules. 2023 Feb 12;28(4):1753. doi: 10.3390/molecules28041753 (PMC9959328; doi:10.3390/molecules28041753)
Supplement: Supplementary file 1 [file molecules-28-01753-s001.zip › molecules-2149474-supplementary.pdf]

molecules

Supplementary Materials

Glucosinolates in Wild-Growing *Reseda* spp. from Croatia

Azra Đulović [1], Josip Tomaš [2] and Ivica Blažević [1,*]

[1] Department of Organic Chemistry, Faculty of Chemistry and Technology, University of Split, Ruđera Boškovića 35, 21000 Split, Croatia

[2] Institute de Chimie Organique et Analytique (ICOA), Université d'Orléans, UMR-CNRS 7311, BP 6759, F-45067 Orléans, France

* Correspondence: blazevic@ktf-split.hr; Tel.: +385-21-329-434

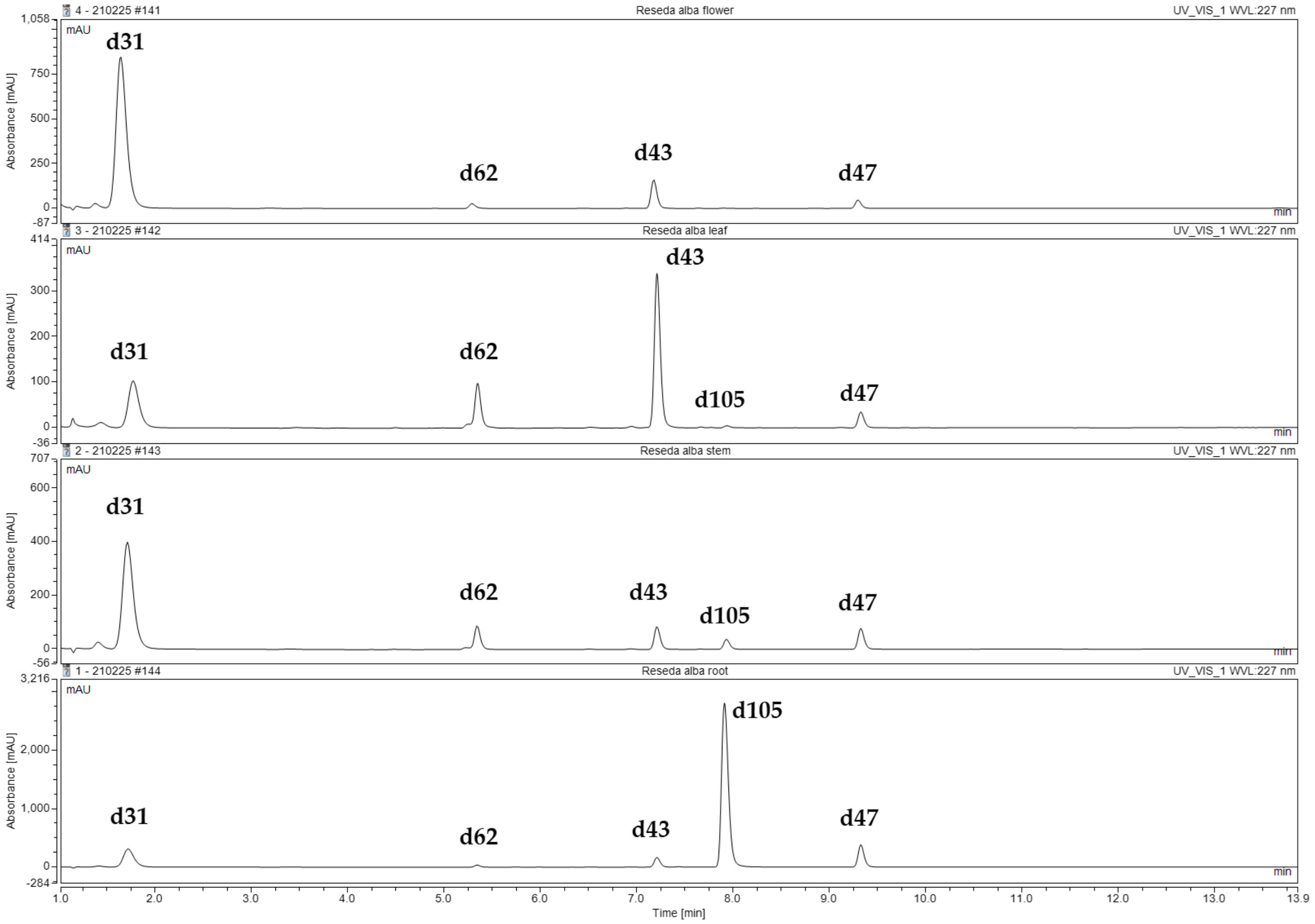

Figure S1. Chromatogram of desulfoglucosinolates obtained from the different plant parts of *Reseda alba* (flower, leaf, stem, root):
d31 – desulfo-2-hydroxy-2-methylpropyl GSL (desulfoglucoconringiin), **d43** - desulfoindol-3-ylmethyl GSL (desulfoglucobrassicin), **d47** – desulfo-*N*-methoxyindol-3-ylmethyl GSL (desulfoneoglucobrassicin), **d62** - desulfoisobutyl GSL, **d105** – desulfo-2-phenylethyl GSL, (desulfogluconasturtiin) .

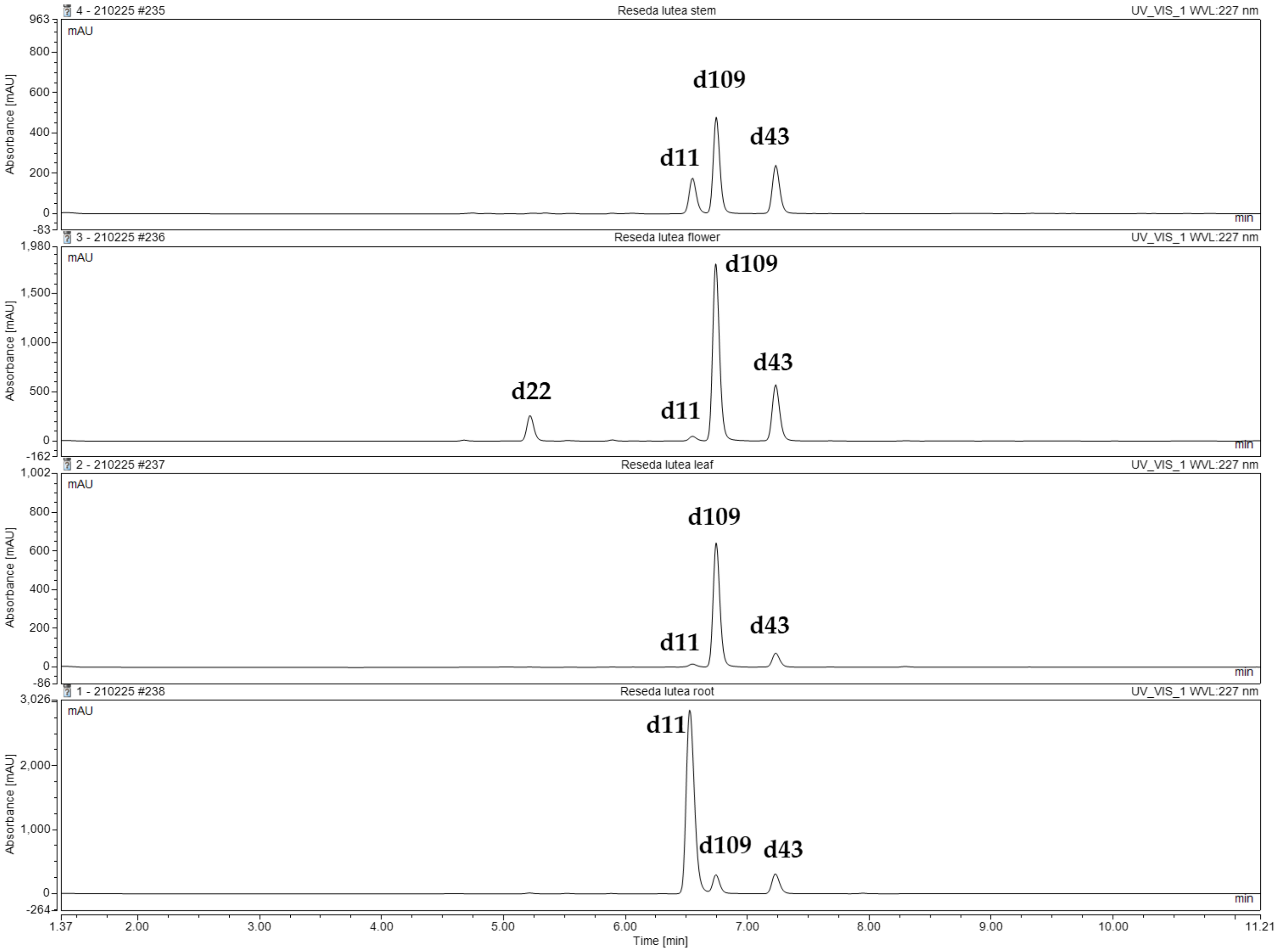

Figure S2. Chromatogram of desulfoglucosinolates obtained from the different plant parts of *Reseda lutea* (stem, flower, leaf, root):
d11 - desulfobenzyl GSL (desulfoglucotropaeolin), **d22** – desulfo-3-hydroxybenzyl GSL (desulfoglucolepigramin), **d43** - desulfoindol-3-ylmethyl GSL (desulfoglucobrassicin), **d109** – desulfo-2-(α-L-rhamnopyranosyloxy)benzyl GSL. .

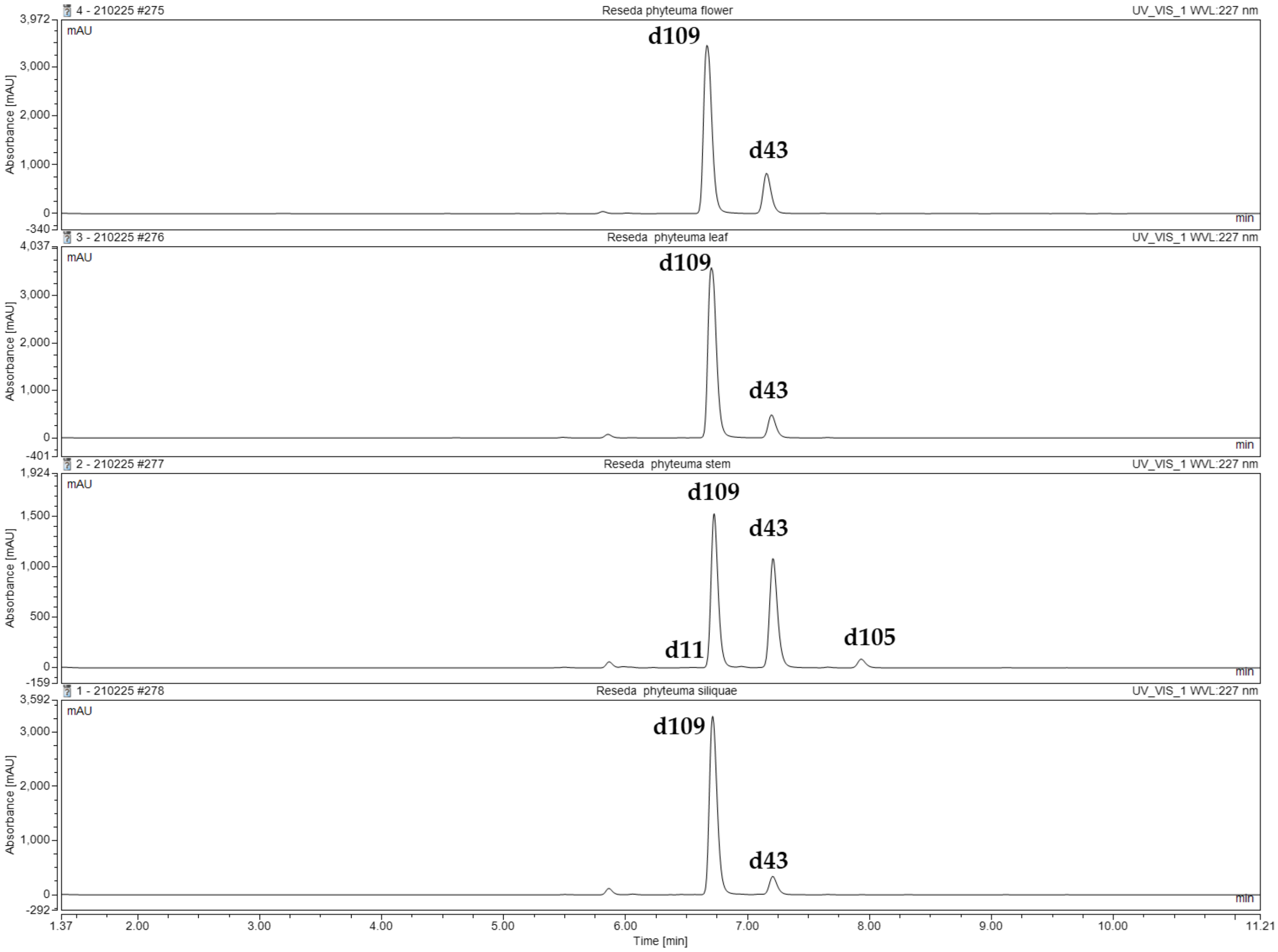

Figure S3. Chromatogram of desulfoglucosinolates obtained from the different plant parts of *Reseda phyteuma* (flower, leaf, stem, siliquae): **d11** - desulfobenzyl GSL (desulfoglucotropaeolin), **d43** - desulfoindol-3-ylmethyl GSL (desulfoglucobrassicin), **d105** – desulfo-2-phenylethyl GSL (desulfogluconasturtiin), **d109** – desulfo-2-(α-L-rhamnopyranosyloxy)benzyl GSL.

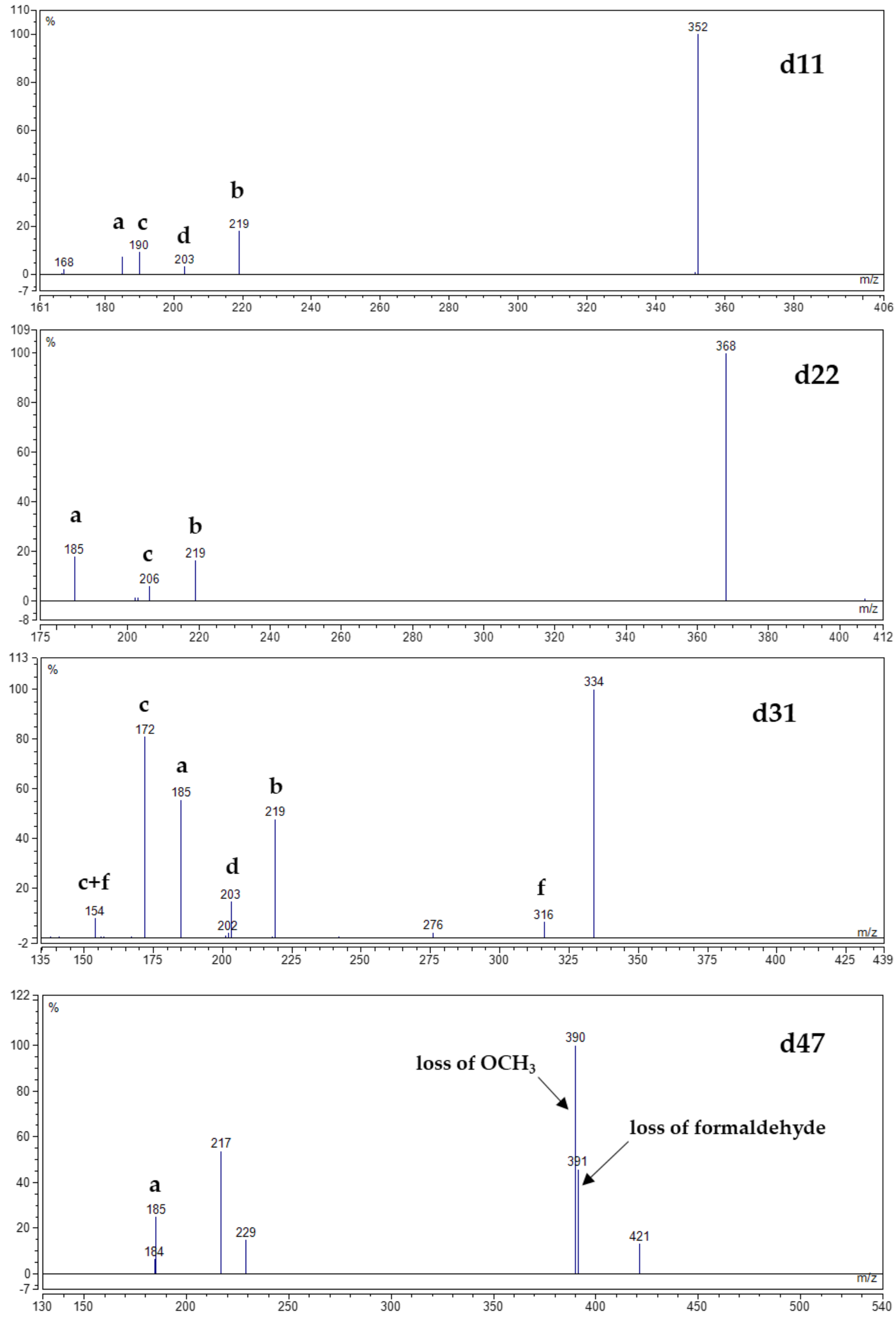

Figure S4A. *Continuous*

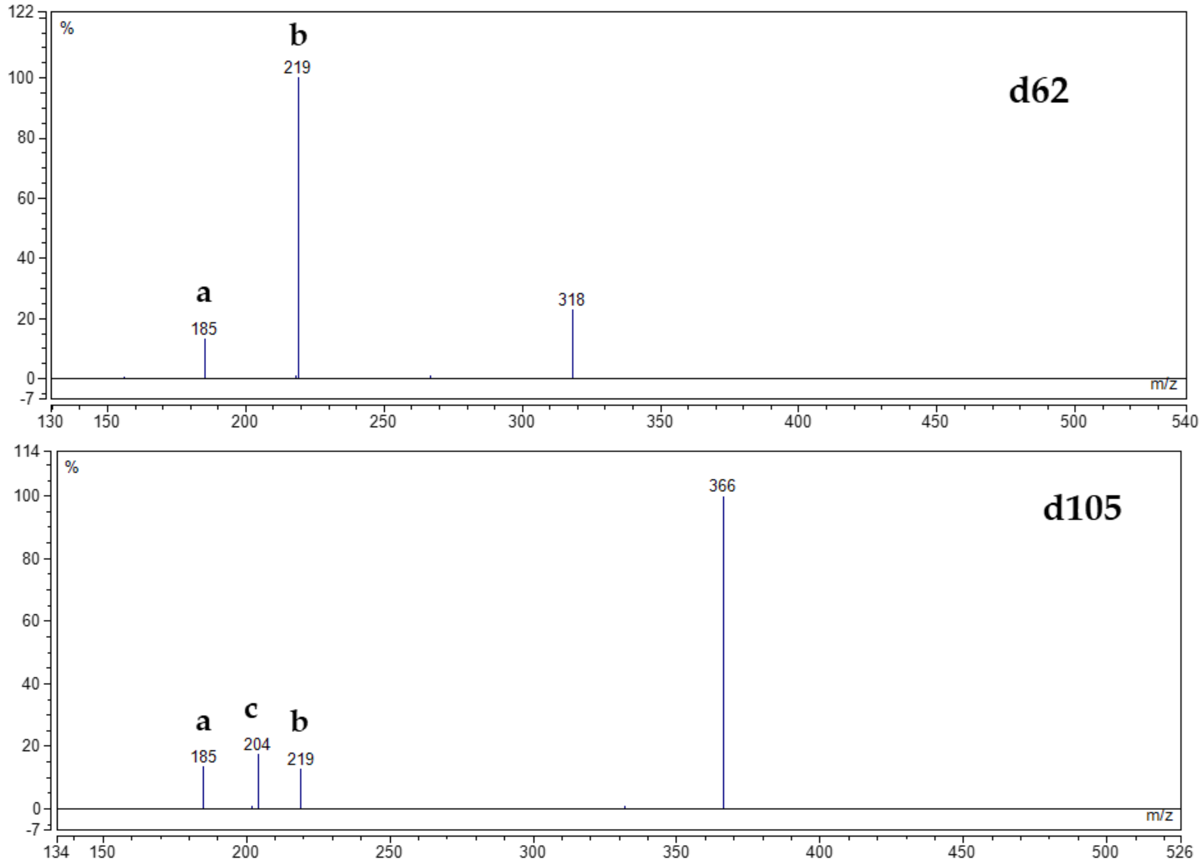

Figure S4. A. MS^2 spectra at 15V of detected desulfoglucosinolates. Fragment types observed, alone or in combination, in MS^2 spectra desulfoglucosinolates (dGSLs) in positive mode: **a** - Na^+ adduct of anhydroglucose, $C_6H_{10}O_5$ (at *m*/*z* 185) or an acyl derivative; **b** - Na^+ adduct of thioglucose, $C_6H_{11}O_5SH$ (at *m*/*z* 219) or an acyl derivative; **c** - Loss of anhydroglucose (*m*/*z* 162) or an acyl derivative; **d** - Na^+ adduct of glucose, $C_6H_{12}O_6$ (at m/z 203); **e** - Loss of thioglucose (*m*/*z* 196) or an acyl derivative; **f** - loss of water.

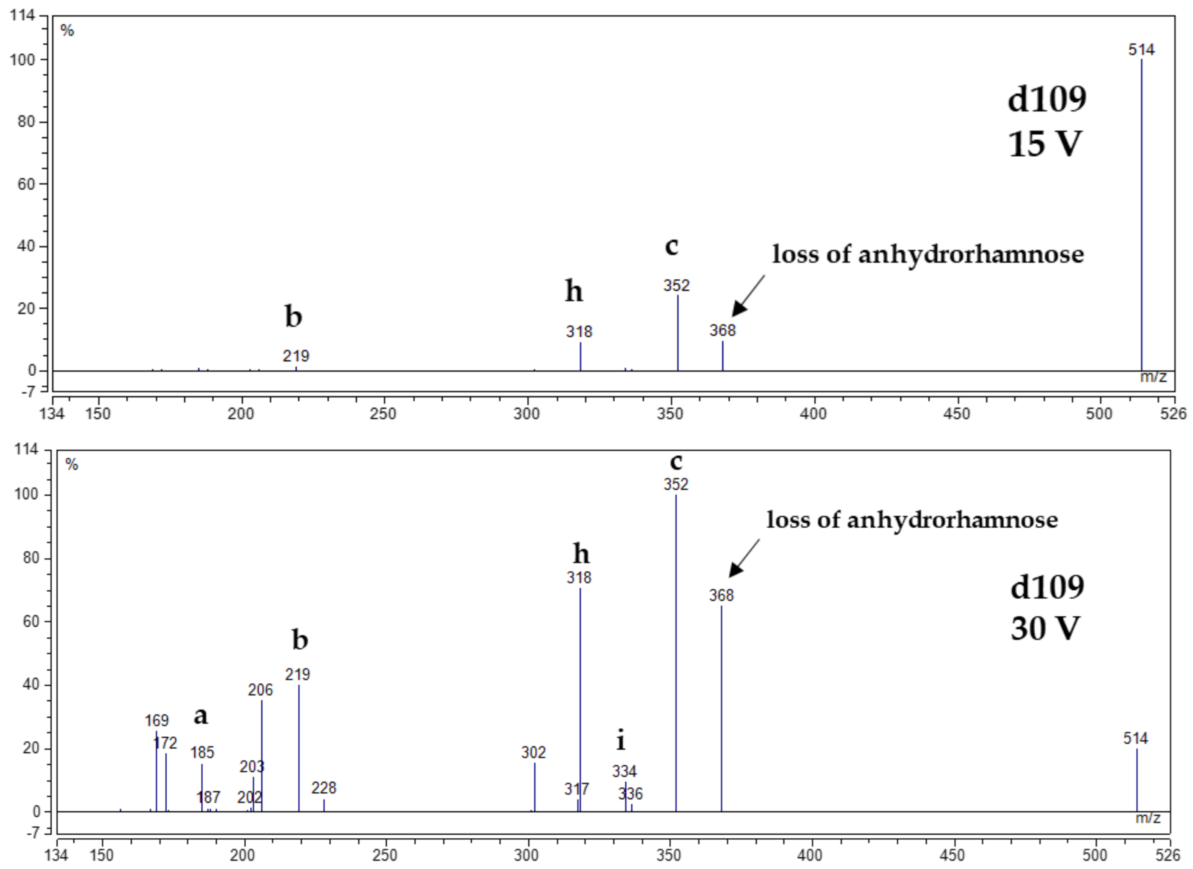

Figure S4. B. MS[2] spectra of **d109** at 20V and 30 V. Fragment types observed, alone or in combination, in MS[2] spectra desulfoglucosinolates (dGSLs) in positive mode: **a**, **b**, **c** as indicated in Figure S4A; **h** - loss of thioglucose (m/z 196) or an acyl derivate; **i** - loss of glucose (m/z 180).

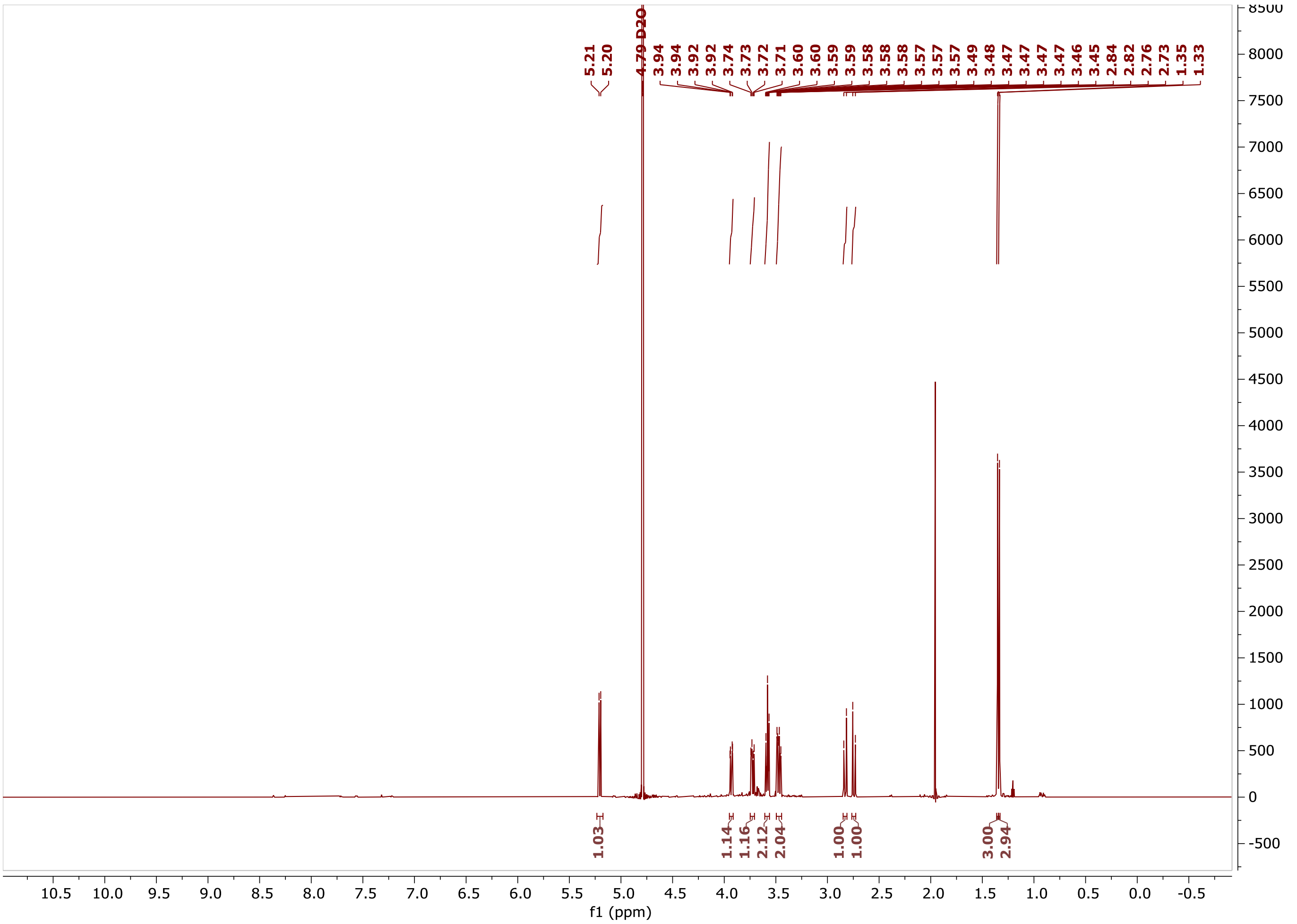

Figure S5A. ^{1}H NMR spectrum of desulfo-2-hydroxy-2-methylpropyl GSL (desulfoglucoconringiin).

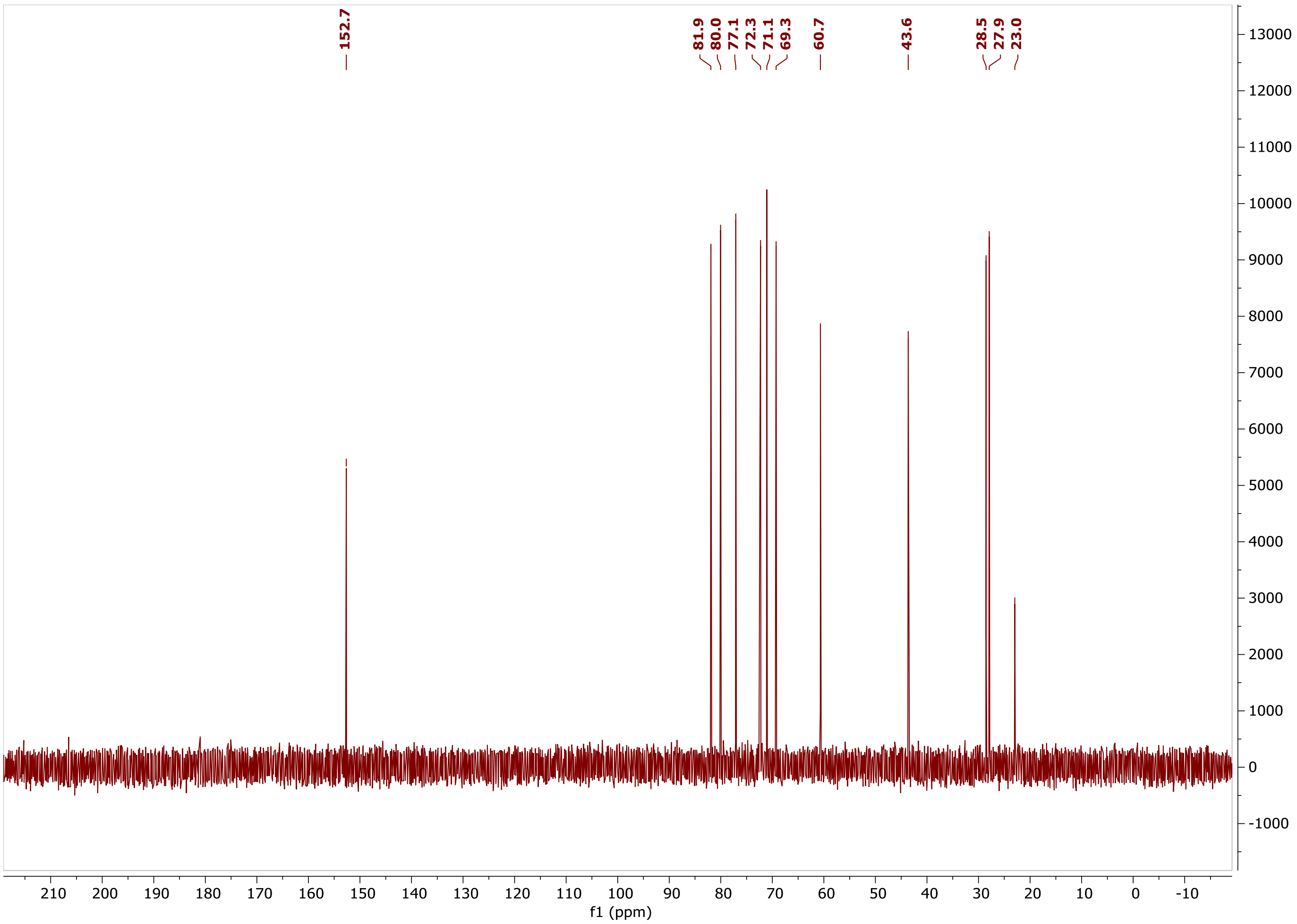

Figure S5B . ^{13}C NMR spectrum of desulfo-2-hydroxy-2-methylpropyl GSL (desulfoglucoconringiin).

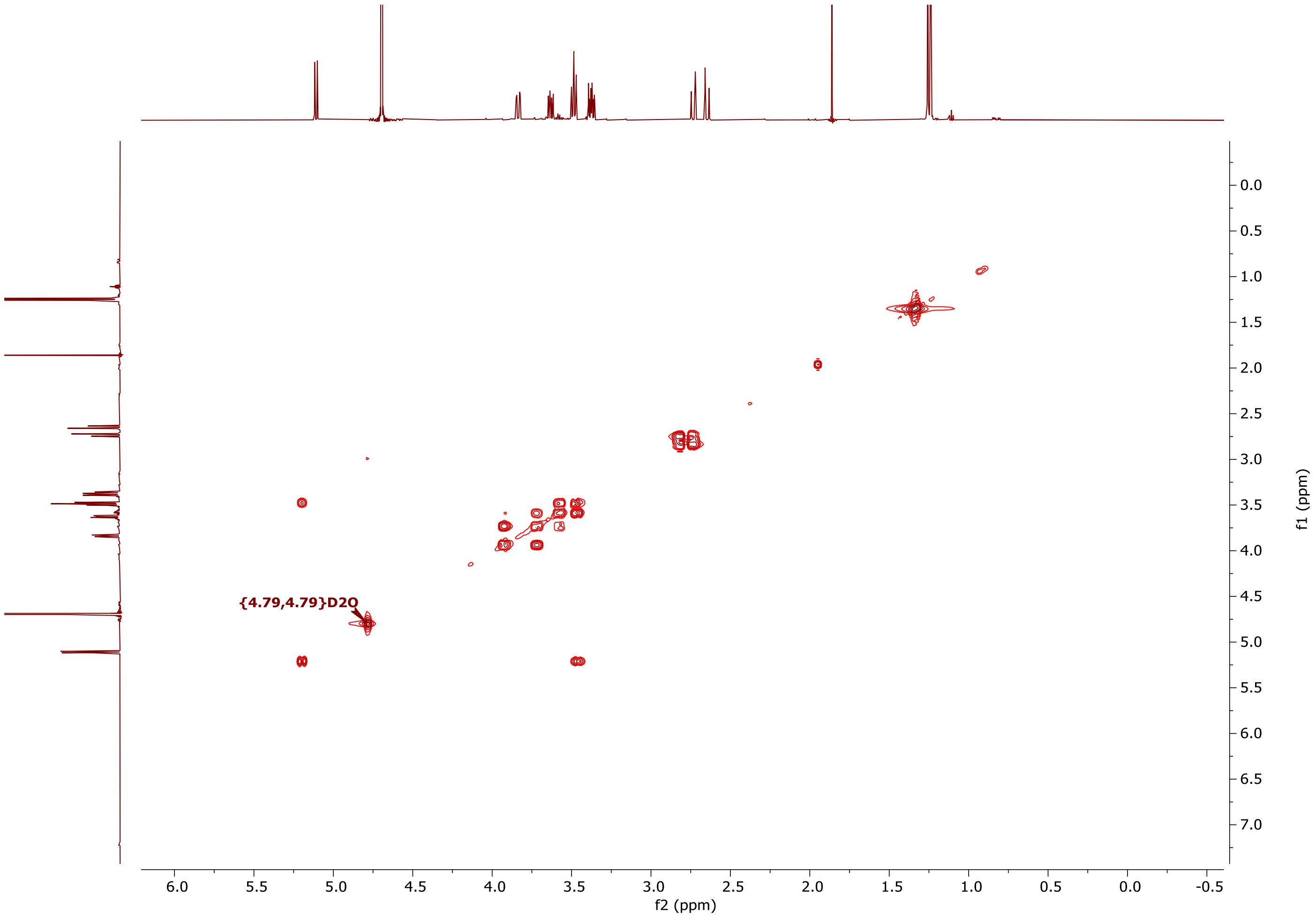

Figure S5C. COSY spectrum of desulfo-2-hydroxy-2-methylpropyl GSL (desulfoglucoconringiin).

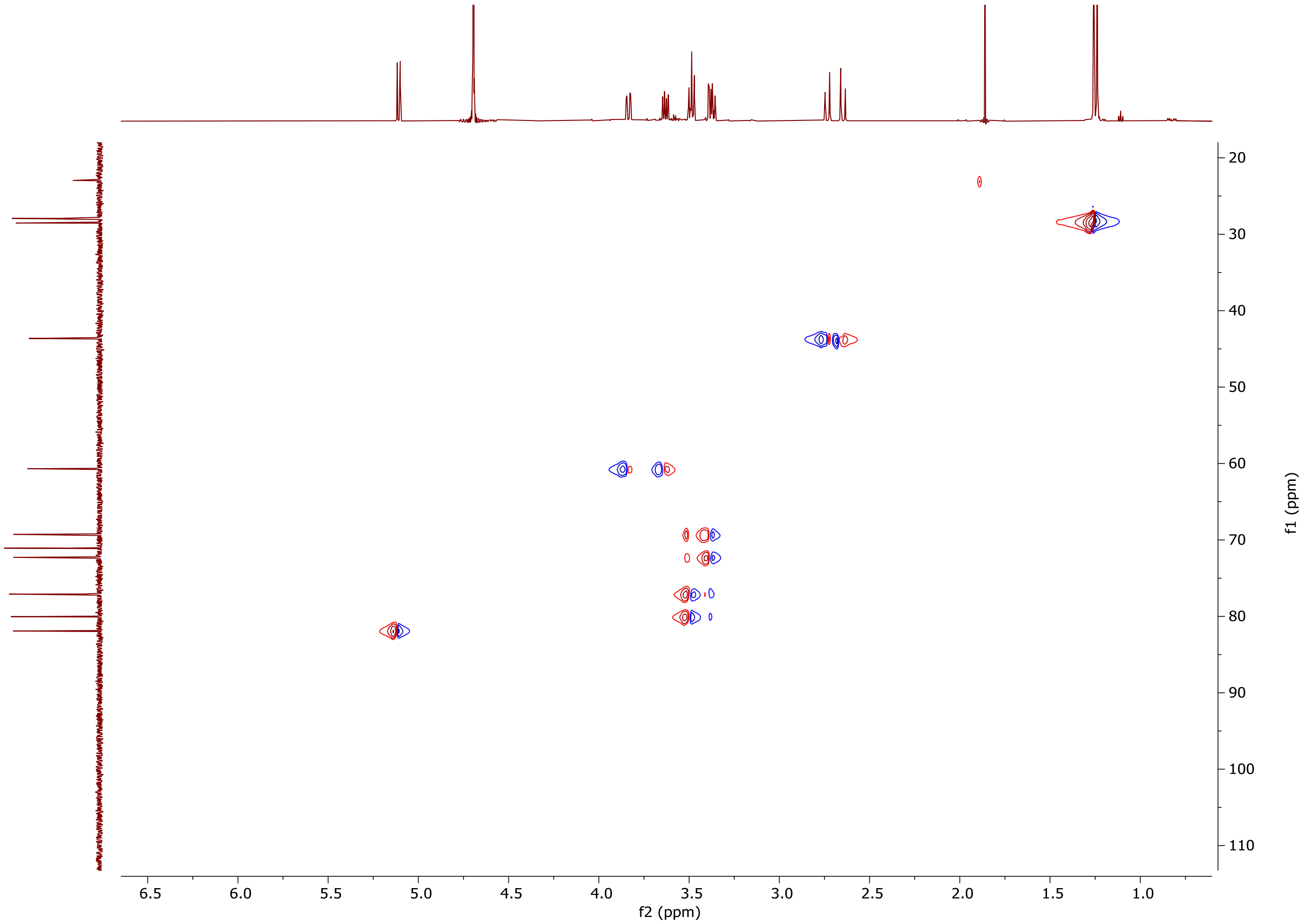

Figure S5D. HSQC spectrum of desulfo-2-hydroxy-2-methylpropyl GSL (desulfoglucoconringiin).
